# Supplementary material for: A Novel Recombinant Fcγ Receptor-Targeted Survivin Combines with Chemotherapy for Efficient Cancer Treatment
Source: Biomedicines. 2021 Jul 12;9(7):806. doi: 10.3390/biomedicines9070806 (PMC8301409; doi:10.3390/biomedicines9070806)
Supplement: Supplementary file 1 [file biomedicines-09-00806-s001.zip › biomedicines-1261602-supplementary.pdf]

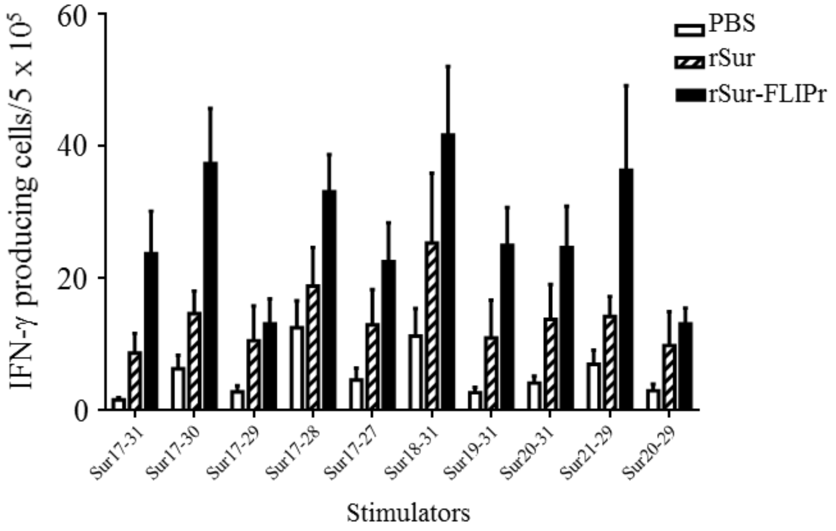

Figure S1. Identification of the epitope within survivn$_{17\text{-}30}$. Groups of C57BL/6 mice (n = 6/group, pooled from two independent experiments) were immunized twice with 30 μg of rSur or rSur-FLIPr at a 2-week interval. Mice immunized with PBS were used as the controls. Splenocytes were prepared 1 week after the last immunization. A panel of peptides that covered the sequence of survivn$_{17\text{-}31}$ was used to stimulate splenocytes for 72 hours in anti-IFN-γ-coated 96-well ELISPOT plates. The IFN-γ-secreting spots were examined using an ELISPOT reader. The data are expressed as the means ± SEM.
